# Genetic effects of fatty acid composition in muscle of Atlantic salmon

**DOI:** 10.1186/s12711-018-0394-x

**Published:** 2018-05-02

**Authors:** Siri S. Horn, Bente Ruyter, Theo H. E. Meuwissen, Borghild Hillestad, Anna K. Sonesson

**Affiliations:** 1 Nofima (Norwegian Institute of Food, Fisheries and Aquaculture Research), PO Box 210, 1432 Ås, Norway; 2 Department of Animal and Aquaculture Sciences, Norwegian University of Life Sciences, 1430 Ås, Norway; 3 SalmoBreed AS, Sandviksboder 3A, 5035 Bergen, Norway

## Abstract

**Background:**

The replacement of fish oil (FO) and fishmeal with plant ingredients in the diet of farmed Atlantic salmon has resulted in reduced levels of the health-promoting long-chain polyunsaturated omega-3 fatty acids (n-3 LC-PUFA) eicosapentaenoic (EPA; 20:5n-3) and docosahexaenoic acid (DHA; 22:6n-3) in their filets. Previous studies showed the potential of selective breeding to increase n-3 LC-PUFA levels in salmon tissues, but knowledge on the genetic parameters for individual muscle fatty acids (FA) and their relationships with other traits is still lacking. Thus, we estimated genetic parameters for muscle content of individual FA, and their relationships with lipid deposition traits, muscle pigmentation, sea lice and pancreas disease in slaughter-sized Atlantic salmon. Our aim was to evaluate the selection potential for increased n-3 LC-PUFA content and provide insight into FA metabolism in Atlantic salmon muscle.

**Results:**

Among the n-3 PUFA, proportional contents of alpha-linolenic acid (ALA; 18:3n-3) and DHA had the highest heritability (0.26) and EPA the lowest (0.09). Genetic correlations of EPA and DHA proportions with muscle fat differed considerably, 0.60 and 0.01, respectively. The genetic correlation of DHA proportion with visceral fat was positive and high (0.61), whereas that of EPA proportion with lice density was negative. FA that are in close proximity along the bioconversion pathway showed positive correlations with each other, whereas the start (ALA) and end-point (DHA) of the pathway were negatively correlated (− 0.28), indicating active bioconversion of ALA to DHA in the muscle of fish fed high FO-diet.

**Conclusions:**

Since contents of individual FA in salmon muscle show additive genetic variation, changing FA composition by selective breeding is possible. Taken together, our results show that the heritabilities of individual n-3 LC-PUFA and their genetic correlations with other traits vary, which indicates that they play different roles in muscle lipid metabolism, and that proportional muscle contents of EPA and DHA are linked to body fat deposition. Thus, different selection strategies can be applied in order to increase the content of healthy omega-3 FAin the salmon muscle. We recommend selection for the proportion of EPA + DHA in the muscle because they are both essential FA and because such selection has no clear detrimental effects on other traits.

**Electronic supplementary material:**

The online version of this article (10.1186/s12711-018-0394-x) contains supplementary material, which is available to authorized users.

## Background

Traditionally, farmed Atlantic salmon (*Salmo salar* L.) were fed diets rich in fish oil (FO) and fishmeal. Limited and decreasing availability of raw materials from wild fisheries have led to the replacement of a large portion of the marine ingredients with more sustainable plant-based ingredients in aquaculture feed [1]. Well-documented consequences of this change are decreased levels of the health-promoting omega-3 long-chain polyunsaturated fatty acids (n-3 LC-PUFA) eicosapentaenoic (EPA; 20:5n-3) and docosahexaenoic acids (DHA; 22:6n-3) in salmon filets, because these fatty acids (FA) are not present in plant oils [2, 3].

Atlantic salmon store most of their energy in the form of lipids in their muscle. The largest portion of such lipids is in the adipocytes along connective tissue sheets known as myocepta [4, 5]. Lipids present in the myocepta have a high proportion of triacylglycerol (TAG), with a FA composition that is strongly influenced by the diet. Thus, muscle lipids have high levels of 18-carbon unsaturated FA 18:1n-9, 18:2n-6 and 18:3n-3 and saturated FA palmitic acid (PA; 16:0) [6]. Another important lipid group in muscle is phospholipids (PL), which are located mainly in cell membranes [7]. Compared to TAG, PL have high levels of n-3 LC-PUFA, and their FA composition is more strictly regulated in order to maintain cell membrane functionality [8, 9]. Fat percentage in the muscle increases with growth and development of the fish [10, 11]. A higher fat content results in an increased ratio of TAG versus PL and, thus, influences FA composition.

Muscle FA composition is strongly influenced by the diet, although not in an entirely linear way [12]. It is a function of several processes, each regulated on a cellular, tissue and whole-body level for maintaining lipid homeostasis [8]. Several studies have reported selective oxidation and deposition of individual FA in salmonids [13–16] and Vegusdal et al. [17] provided evidence of preferential uptake of specific FA into the muscle. Another process that influences the FA composition is bioconversion. Salmonids can convert the shorter-chained FA alpha-linolenic acid (ALA; 18:3n-3), which is commonly found in some plant oils, into longer chained EPA and DHA [18, 19]. This metabolic pathway was first described in mammals by Sprecher [20] and consists of a series of desaturation and elongation reactions. The first step is Δ6 desaturation of 18:3n-3 to produce 18:4n-3 that is elongated to 20:4n-3. Alternatively, 18:3n-3 is elongated to 20:3n-3 followed by a Δ8 desaturation. 20:4n-3 is desaturated by Δ5 desaturase to form EPA [21, 22]. DHA synthesis from EPA requires two more elongation steps, a second Δ6 desaturation, and a chain-shortening step by peroxisomal β-oxidation [23]. Activity of the desaturase and elongase enzymes determines the relative amounts of EPA and DHA formed. Several factors influence the activities of these enzymes: nutrition, environment, hormones and genetics. In salmonids, liver, intestinal and muscle cells can convert ALA to DHA [24, 25]. Thus, this bioconversion is expected to influence the muscle FA composition, although less than the diet.

There is evidence that genetics affects muscle FA composition in Atlantic salmon [26]; Schlechtriem et al. [27] reported individual variation in the content of n-3 PUFA in the flesh of Atlantic salmon fed the same feed, which indicates a strong genetic influence on this trait. Similarly, Leaver et al. [28] found that the content of total n-3 LC-PUFA in salmon muscle differed between families and was highly heritable. Selection for increased liver expression of genes encoding enzymes in the bioconversion pathway has led to increased levels of DHA in the liver of salmon [29]. Hence, there is potential in using selective breeding as a tool to increase levels of n-3 LC-PUFA in Atlantic salmon muscle by increasing the salmon’s natural capacity to convert the shorter-chained 18:3n-3 FA from plant oils into n-3 LC-PUFA and to efficiently deposit these FA in the muscle. However, knowledge on the genetic parameters of individual muscle FA and their relationships with lipid deposition traits and other breeding traits (e.g. carcass, quality and disease) is lacking, but is key to predicting the consequences of selection for higher n-3 LC-PUFA levels. Thus, the objective of this study was to estimate genetic parameters of individual FA in the muscle of farmed Atlantic salmon to evaluate the potential for selection for increased n-3 LC-PUFA levels and provide insight into the muscle’s FA metabolism.

## Methods

### Fish populations and trait recording

#### Slaughter test

The data represented one year-class of the Atlantic salmon breeding population of SalmoBreed AS. The fish were transferred to net pens in the sea at a mean weight of 113.1 g and harvested at a mean slaughter weight of 3605 g. In total, 668 fish that were reared under the same conditions were included in this study. They were fed a commercial broodstock feed with a high FO content (see Additional file 1) and were fasted 13 to 14 days prior to slaughter. These fish came from 194 full-sib families that originated from 92 sires and 194 dams. All sires had four or more offspring from more than one dam.

The following traits were recorded at slaughter: body weight (g); length (cm); sex, determined visually by inspection of the gonads; muscle pigment (mg/kg), the carotenoid astaxanthin was measured immediately after slaughter using a commercial NIR imaging scanner (Tomra Sorting Solutions, Leuven, Belgium) [30, 31]; liver fat, an indicator of the degree of fatty liver, determined visually on a scale of 1 (darkest color i.e. healthy) to 5 (lightest color i.e. fatty liver), which is reversed compared to the scale presented in [32] to facilitate interpretation of the results; visceral fat, determined visually on a scale of 1 (lowest amount of fat) to 5 (highest) [32].

Muscle samples from the Norwegian Quality Cut (NQC) of the fillet were collected at harvest from each fish, frozen, and stored at − 20 °C.

#### Sea lice and pancreas disease challenge tests

Data from sea lice and pancreas disease (PD) challenge tests of siblings of the analyzed fish were obtained from SalmoBreed AS, as part of their larger challenge tests of the 2014 year class.

In the sea lice challenge test, 2207 post-smolts (body weight ~ 60 g) were infected by mixing sea lice copepodites in a water basin (12 °C) with an infection rate of about 30 copepodites per fish. The sea lice strain was LS Gulen from 2006. The fish were sedated and the number of lice per fish was counted after sea lice reached the motile stage (~ 14 days) (personal communication Borghild Hillestad). Sea lice density was calculated as lice count/body weight^2/3^, following [33].

In the PD challenge test, 2426 post-smolts (body weight ~ 65 g) were infected with the SAV3 virus by direct intraperitoneal injection, following the VESO Vikan protocol that was approved by the Norwegian Animal Research Authority (personal communication Borghild Hillestad). The total PD survival rate at the end of the challenge was 60%.

### Lipid and fatty acid analyses

Total lipids were extracted from homogenized NQC muscle samples of individual fish sampled at slaughter, according to the method described by Folch et al. [34]. Using 1 mL of the chloroform–methanol phase, FA composition of the total lipids was analyzed according to the method described by Mason and Waller [35]. The extract was dried quickly under nitrogen gas and the residual lipid extract was trans-methylated overnight with 2,2-dimethoxypropane, methanolic-HCl, and benzene at room temperature. The methyl esters formed were separated in a gas chromatograph with a split injector, using an SGE BPX70 capillary column (length 60 m, internal diameter 0.25 mm, and film thickness 0.25 μm; SGE Analytical Science, Milton Keynes, UK) and a flame ionization detector. The results were analyzed using the HP Chem Station software (Hewlett Packard 6890; HP, Wilmington, DE, USA). The carrier gas was helium, and both injector and detector temperatures were 270 °C. The oven temperature was raised from 50 to 170 °C at a rate of 4 °C/min and then raised to 200 °C at a rate of 0.5 °C/min and finally to 240 °C at a rate of 10 °C/min. Individual FA methyl esters were identified by reference to well-characterized standards. The proportional content of each FA was expressed as a percentage of the total amount of FA in the analyzed sample. Absolute content of each FA was calculated as described in Folch et al. [34]: FA in g per 100 g muscle = (% FA of total FA/00) × (Muscle fat % × 0.9).

Presentation of the results will focus on the most abundant FA in the fillet (16:0, 18:1n-9, 18:2n-6) and the following FA that are part of the bioconversion pathway: 18:3n-3, 20:3n-3, 20:4n-3, 20:5n-3, 22:5n-3 and 22:6n-3.

### Statistical analyses

Variance and covariance components were estimated by residual maximum likelihood procedures using the ASReml Package [36]. Bivariate analyses were performed to estimate genetic correlations between traits, using the following bivariate animal model [37]:

$${\mathbf{Y}} = {\mathbf{XB}} + {\mathbf{U}} + {\mathbf{E}},$$

where ${\mathbf{Y}}$ is a matrix of phenotypic records for individuals *i* = 1, 2, …, *n* and traits *j* = 1, 2, ${\mathbf{X}}$ is a matrix of the fixed effects of animal *i* on trait *j*, ${\mathbf{B}}$ is a matrix of fixed effect solutions. ${\mathbf{U}}$ is a matrix containing the random effects of animal *i* on trait *j*, with variance ${\mathbf{G}} \otimes {\mathbf{A}}$, where ${\mathbf{A}}$ is the relationship matrix between individuals and ${\mathbf{G}}$ is a genetic variance–covariance matrix among traits. ${\mathbf{E}}$ is a matrix of residual effects, that is assumed to have a variance of $${\mathbf{R}} = \left[ {\begin{array}{*{20}c} {\sigma_{e1}^{2} } & {\sigma_{e1, e2} } \\ {\sigma_{e1, e2} } & {\sigma_{e2}^{2} } \\ \end{array} } \right]$$, where 1 and 2 indicate traits. Univariate analyses were performed to estimate heritabilities for all traits. For the univariate analyses, matrices ${\mathbf{Y}}$, ${\mathbf{B}}$, ${\mathbf{U}}$ and ${\mathbf{E}}$ were reduced to vectors and matrices ${\mathbf{G}}$ and ${\mathbf{R}}$ were reduced to scalars. Heritability estimates (*h*^2^) were calculated as the ratio of additive genetic ($\upsigma_{\text{A}}^{2}$) to total phenotypic ($\upsigma_{\text{P}}^{2}$) variance ($h^{2} =\upsigma_{\text{A}}^{2} /\upsigma_{\text{P}}^{2}$). Correlations between two traits (*r*) were calculated as the covariance divided by the square root of the product of two variances i.e. $r = \text{cov}_{1,2} /\sqrt {\upsigma_{1}^{2} \times\upsigma_{2}^{2} }$.

Body weight was included as a covariate and sex was included as a fixed effect for FA, pigment, visceral fat, liver fat and muscle fat. For PD survival, cage was included as random effect. For lice density, the person performing the counting and the day of recording were included as fixed effects and cage was included as a random effect. Four generations of pedigree information on direct ancestors of the fish in the tests were available (n = 11,801). Estimates were considered as significantly different from zero if they deviated more than two times their standard error from zero (*p* < 0.05).

## Results

### Data description

The coefficients of variation (CV) showed that traits varied extensively among the sampled fish (Table 1), especially body weight, visceral fat and liver fat, which each had a CV above 20%. The average fat content of NQC muscle samples was 19% (Table 1), which is high for fish of this size but not extreme. The average liver fat score was within the normal range (2.13).

**Table 1:** Descriptive statistics for body size, fat deposition, fillet quality and disease traits

Trait category	Trait	N	Mean	SD	Min	Max	CV (%)
Body size	Body weight (g)	668	3605	861	1150	6380	24
	Length (cm)	668	66.5	5.1	50	78	8
Fat deposition	Muscle fat (%)	668	19.07	3.16	5.47	27.65	17
	Visceral fat (1–5)	668	3.40	0.83	1	5	24
	Liver fat (1–5)	668	2.13	0.76	1	5	26
Fillet quality	Pigment (mg/kg muscle)	668	7.7	0.9	3.9	10.7	11
Disease	Sea lice density^a^	2207	64.25	26.25	0	202.98	41
	PD survival^b^	2426	0.62	–	0	1	–

*N* number of observations, *SD* standard deviation, *Min* minimum value, *Max* maximum value, *CV* coefficient of variation (SD/mean × 100)

^a^Sea lice density = lice count/body weight^2/3^

^b^Scored 0 or 1, where 0 is dead and 1 is alive after the challenge test

The major muscle FA were 18:1n-9, 18:2n-6 and the saturated FA 16:0 (Table 2). The mean muscle contents of EPA and DHA were approximately twice as high as in commercially-produced salmon, which reflects the high level of these FA in the feed (see Additional file 1). The absolute content of individual muscle FA varied greatly, as shown by their CV, which ranged from 15 to 33%, with EPA showing the largest variation. The muscle content of each individual FA was approximately normally distributed. The distributions of proportional contents of individual FA in the muscle across fish are in Additional file 2. The FA that are not shown in Table 2 amounted to approximately 30% of total FA.

**Table 2:** Descriptive statistics of proportional and absolute content of selected fatty acids in muscle

Fatty acid		N	Proportional content (% of total FA)			Absolute content (g FA/100 g muscle)		
			Mean	SD	CV (%)	Mean	SD	CV (%)
16:0	Palmitic acid (PA)	668	11.69	0.71	6	2.01	0.38	19
18:1n-9	Oleic acid (OA)	668	30.45	1.91	6	5.22	0.90	17
18:2n-6	Linoleic acid (LA)	668	9.65	0.73	8	1.65	0.27	16
18:3n-3	Alpha-linolenic acid (ALA)	668	3.46	0.23	7	0.59	0.10	17
20:3n-3	Eicosatrienoic acid	668	0.36	0.05	13	0.06	0.01	23
20:4n-3	Eicosatetraenoic acid	634	0.54	0.16	30	0.09	0.03	33
20:5n-3	Eicosapentaenoic acid (EPA)	668	5.42	1.01	19	0.93	0.24	25
22:5n-3	Docosapentaenoic acid (DPA)	668	2.36	0.21	9	0.41	0.08	19
22:6n-3	Docosahexaenoic acid (DHA)	668	6.75	0.51	8	1.15	0.18	15
	Sum EPA + DHA	668	12.17	1.26	10	2.08	0.38	18

*N* number of observations, *SD* standard deviation, *CV* coefficient of variation (SD/Mean × 100)

**Fig. 1:**
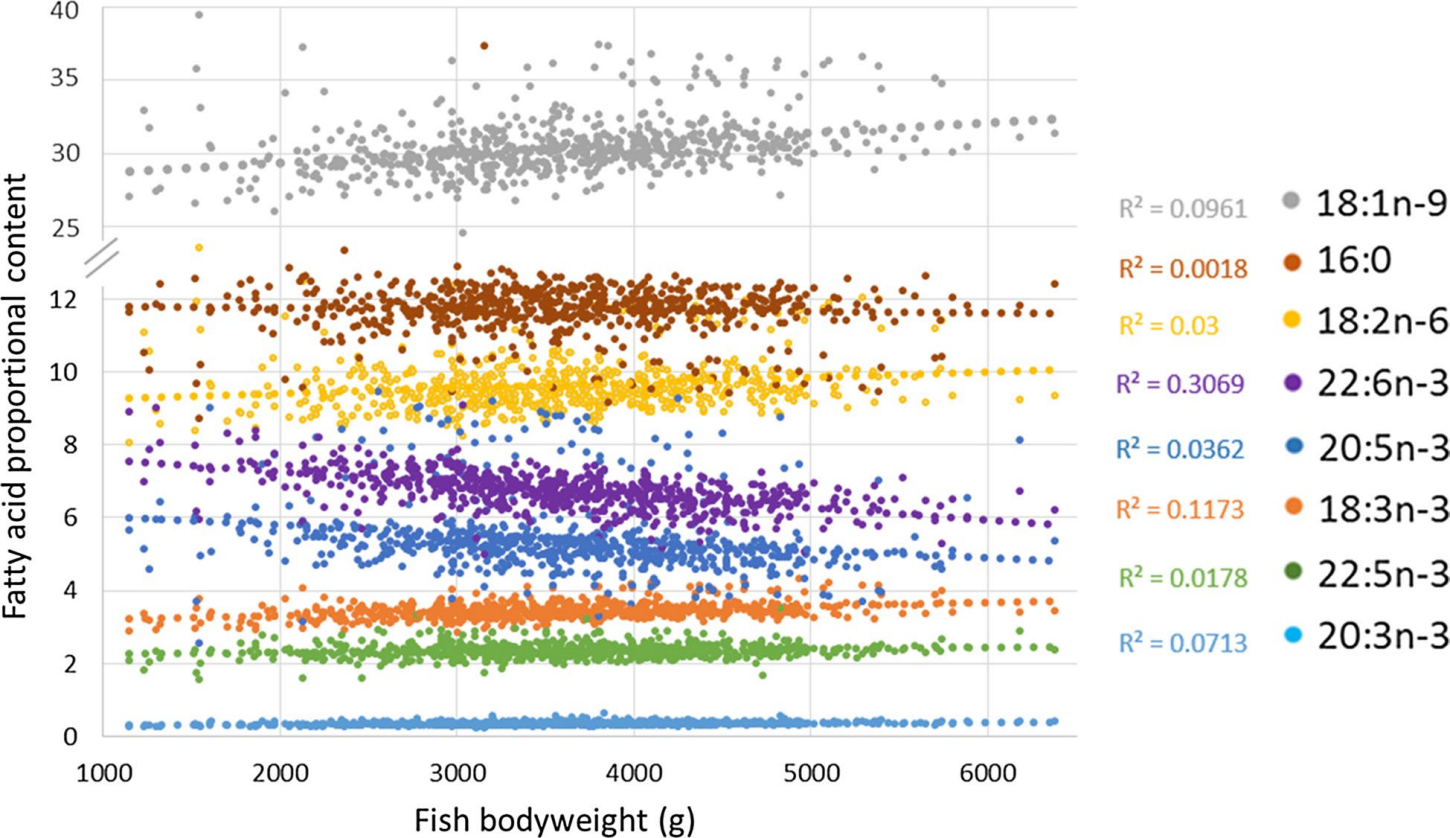
Relationship between fish body weight and proportional content of fatty acids in the muscle

The proportional contents of muscle FA changed with increasing body weight (Fig. 1). The content of PA (16:0) remained stable, whereas that of 18-carbon FA increased and those of EPA and DHA were the only ones that decreased with increasing body weight. In the remaining results, all estimates were corrected for body weight.

### Metabolism

The heritability of the absolute content of the n-3 PUFA, in g per 100 g muscle, was generally moderate to high, which reflects the high heritability of muscle fat (0.46) (see Additional file 3). DHA had the highest heritability (0.46) (Table 3). The absolute content of EPA + DHA had a heritability of 0.35 (Table 3), whereas proportional content had a heritability of 0.09 (SE 0.06). For proportional contents of FA, the start and end-point of the bioconversion pathway (ALA and DHA) had the highest heritabilities, whereas EPA had the lowest heritability (0.09, Table 4).

**Table 3:** Estimates of heritability for absolute content of selected fatty acids

FA (g)	Heritability (SE)
18:3n-3	0.34 (0.09)
20:3n-3	0.23 (0.08)
20:4n-3	0.17 (0.08)
20:5n-3	0.25 (0.07)
22:5n-3	0.37 (0.09)
22:6n-3	0.46 (0.09)
EPA + DHA	0.35 (0.09)
Ratio DHA:ALA	0.20 (0.08)

FA (g): muscle fatty acid content in grams per 100 grams of muscle

Standard errors in brackets

**Table 4:** Estimates of heritability and of genetic and phenotypic correlations for proportional content of selected fatty acids

FA (%)	18:3n-3	20:3n-3	20:4n-3	20:5n-3	22:5n-3	22:6n-3
18:3n-3	*0.26 (0.08)*	0.21 (0.04)	− 0.21 (0.04)	^a^	− 0.15 (0.04)	− 0.56 (0.03)
20:3n-3	− 0.03 (0.28)	*0.18 (0.08)*	− 0.20(0.04)	0.40 (0.03)	0.43 (0.03)	− 0.06 (0.04)
20:4n-3	0.03 (0.31)	0.07 (0.33)	*0.14 (0.07)*	− 0.33 (0.04)	− 0.14 (0.04)	0.25 (0.04)
20:5n-3	^a^	0.01 (0.42)	− 0.21 (0.40)	*0.09 (0.06)*	0.69 (0.02)	0.23 (0.04)
22:5n-3	− 0.44 (0.23)	0.30 (0.25)	0.19 (0.32)	0.42 (0.26)	*0.22 (0.07)*	0.32 (0.04)
22:6n-3	− 0.28 (0.22)	0.33 (0.28)	0.64 (0.25)	0.16 (0.34)	0.41 (0.21)	*0.26 (0.08)*

FA (%): muscle fatty acid content in percentage of total muscle fatty acids. Heritability on the diagonal. Phenotypic correlations on the upper triangle. Genetic correlations on the lower triangle. Standard errors in brackets

^a^Parameters not converged

Estimates of genetic and phenotypic correlations both showed the same pattern: contents of FA that were in close proximity in the pathway had positive correlations with each other, whereas correlations between contents of the start- (ALA) and end-points (DHA) of the pathway were negative (Table 4). The genetic correlation between the contents of 20:4n-3 and DHA was high.

### Correlations between fatty acid contents and fat deposition traits

Muscle fat had positive phenotypic correlations with proportional contents of PA and EPA (Table 5), but a negative correlation with the proportional contents of the 18-carbon FA. Phenotypic correlations of proportional contents of DPA (22:5 n-3) and DHA with muscle fat were close to zero. Estimates of genetic correlations between muscle fat and proportional contents of FA showed the same pattern, but were higher than the phenotypic correlations, except for the proportional contents of the very long-chain (VLC) (≥ C_22_) PUFA DPA and DHA, which had genetic correlations of nearly zero with muscle fat when correcting for body weight. As expected, genetic correlations between absolute amounts of all FA and muscle fat were highly positive, because an increase in muscle fat increases the absolute amount of all FA (see Additional file 4), and because muscle fat percentage was used to calculate the absolute amount of FA.

**Table 5:** Estimates of phenotypic and genetic correlations of the proportional content of selected muscle fatty acids with muscle, visceral and liver fat

FA (%)	Muscle fat		Visceral fat		Liver fat	
	r_P_	r_G_	r_P_	r_G_	r_P_	r_G_
16:0	0.44 (0.03)	0.86 (0.10)	0.43 (0.03)	0.66 (0.24)	0.12 (0.04)	0.20 (0.27)
18:1n-9	− 0.38 (0.05)	− 0.67 (0.15)	− 0.41 (0.03)	− 0.67 (0.27)	− 0.14 (0.04)	− 0.17 (0.28)
18:2n-6	− 0.46 (0.03)	− 0.78 (0.11)	− 0.47 (0.03)	− 0.63 (0.24)	− 0.14 (0.04)	− 0.23 (0.26)
18:3n-3	− 0.35 (0.04)	− 0.72 (0.15)	− 0.41 (0.03)	− 0.67 (0.26)	− 0.11 (0.04)	− 0.23 (0.28)
20:3n-3	− 0.01 (0.04)	− 0.28 (0.22)	− 0.02 (0.04)	0.25 (0.46)	0.10 (0.03)	0.02 (0.33)
20:4n-3	0.01 (0.04)	− 0.12 (0.25)	0.11 (0.04)	0.82 (0.40)	0.06 (0.04)	0.79 (0.31)
20:5n-3	0.21 (0.04)	0.60 (0.28)	0.12 (0.04)	− 0.17 (0.51)	0.10 (0.04)	− 0.31 (0.41)
22:5n-3	0.05 (0.04)	0.02 (0.21)	0.01 (0.04)	0.20 (0.38)	0.11 (0.04)	0.19 (0.28)
22:6n-3	− 0.06 (0.04)	0.01 (0.21)	0.24 (0.04)	0.61 (0.37)	0.10 (0.04)	0.32 (0.27)
EPA + DHA	0.14 (0.04)	0.58 (0.32)	0.19 (0.04)	0.36 (0.52)	0.11 (0.04)	− 0.04 (0.42)
Ratio DHA:ALA	0.11 (0.04)	0.38 (0.22)	0.34 (0.04)	0.98 (0.35)	0.11 (0.04)	0.35 (0.29)

FA (%): muscle fatty acid content in percentage of total muscle fatty acids

Standard errors in brackets

*r*_*P*_ phenotypic correlations, *r*_*G*_ genetic correlations

Estimates of genetic and phenotypic correlations of visceral fat correlations with FA reflected those with muscle fat for all FA except for EPA, 20:4n-3, and DHA (Table 5). Proportional contents of both EPA and PA had positive genetic correlations with muscle fat; PA also had a positive genetic correlation with visceral fat (0.66) but EPA had a negative correlation with visceral fat (− 0.17). DHA had a high positive genetic correlation with visceral fat (0.61).

Correlations of FA with liver fat were weaker than those with visceral fat but showed the same pattern (Table 5). The genetic correlations for liver fat were similar to the phenotypic correlations, except for EPA, which had a positive phenotypic but a negative genetic correlation with liver fat.

Overall, Table 5 shows that, for fish at the same age and weight, a fatter muscle had a higher absolute content but lower proportion of the 18-carbon FA and higher absolute and proportional contents of EPA and PA, than a leaner muscle. The proportional content of DHA did not increase with increasing muscle fat at a constant body weight.

### Correlations of fatty acid contents and fat deposition traits with other traits

PD survival had a negative genetic correlation with muscle fat (− 0.29) but, surprisingly, a positive genetic correlation with liver fat (0.28, Table 6). The correlations between PD survival and the proportional contents of FA were generally weak. The estimate of heritability for sea lice density was 0.21. Proportional contents of EPA and DPA had negative genetic correlations with sea lice density. The proportional content of all other FA had close to zero genetic correlations with sea lice density. Pigment was positively correlated with all three fat deposit traits. Only the proportional content of the saturated FA PA had a positive correlation with pigment, while the proportional contents of all unsaturated FA had negative correlations with pigment. These correlations with pigment reflected the correlations of muscle fat with the proportional contents of FA in Table 5, except for EPA and DHA.

**Table 6:** Estimates of genetic correlations of quality and disease traits with fat deposit traits and muscle fatty acids

Trait	PD survival	Sea lice density	Pigment
Heritability	0.28 (0.05)	0.21 (0.04)	0.29 (0.08)
	r_G_	r_G_	r_G_
Muscle fat	− 0.29 (0.15)	− 0.22 (0.16)	0.47 (0.17)
Visceral fat	0.16 (0.20)	0.10 (0.21)	0.44 (0.34)
Liver fat	0.28 (0.20)	0.21 (0.20)	0.50 (0.27)
16:0 (%)	− 0.17 (0.18)	0.07 (0.18)	0.36 (0.21)
18:1n-9 (%)	0.12 (0.19)	0.06 (0.20)	− 0.14 (0.23)
18:2n-6 (%)	0.18 (0.17)	0.06 (0.19)	− 0.27 (0.21)
18:3n-3 (%)	0.08 (0.18)	0.10 (0.20)	− 0.19 (0.22)
20:3n-3 (%)	0.30 (0.21)	− 0.33 (0.22)	− 0.18 (0.29)
20:4n-3 (%)	− 0.06 (0.23)	0.26 (0.23)	0.02 (0.29)
20:5n-3 (%)	− 0.13 (0.28)	− 0.47 (0.28)	− 0.09 (0.33)
22:5n-3 (%)	0.05 (0.18)	− 0.40 (0.19)	− 0.35 (0.23)
22:6n-3 (%)	− 0.02 (0.18)	0.09 (0.19)	− 0.29 (0.23)
EPA + DHA	− 0.11 (0.27)	− 0.39 (0.29)	− 0.26 (0.34)
Ratio DHA:ALA	− 0.07 (0.20)	0.00 (0.22)	− 0.13 (0.26)

*r*_*G*_ genetic correlations

Standard errors in brackets. Fatty acids in proportion of total muscle fatty acids

## Discussion

Genetic parameters of individual muscle FA and their correlations with lipid deposition traits and other traits (carcass, quality, and disease traits) were estimated for 668 slaughter-sized (3.6 kg) Atlantic salmon that were fed a high FO-diet. Our aim was to evaluate the selection potential for increased long-chain omega-3 polyunsaturated FA and provide insight into FA metabolism in salmon muscle.

### Heritability estimates

The results showed presence of additive genetic variation in the individual FA contents of salmon muscle, thus changing FA composition by selective breeding is possible. For several FA, both absolute and proportional contents had a high heritability (Tables 3 and 4). The absolute and proportional contents of DHA had heritabilities of 0.46 and 0.26, respectively. The start- and end-points of the n-3 bioconversion pathway (ALA and DHA) had higher heritabilities than the intermediate FA (20:3n-3, 20:4n-3, 20:5n-3 and 22:5n-3). A possible explanation for this is that the levels of intermediate FA may not be stable since they are shuttled through the pathway. In addition, since contents of these FA are very low, errors in their measurement increase. The proportional content of EPA was high (5.42%) but had the lowest heritability of all FA (0.09) (Tables 2 and 4), which may be explained by its many metabolic roles in the body, i.e. it is converted to DHA, used for eicosanoid synthesis, or directed to produce energy [38]. Thus, the “pool” of EPA is highly variable over time [39], which was reflected by the high CV of EPA content, compared to that of the other FA (Table 2).

Overall, the results of this study show that additive genetic variation can be exploited to change the muscle FA composition by selective breeding. This agrees with studies in both pigs [40] and Atlantic salmon [28] that showed that n-3 LC-PUFA content in muscle has a heritable component. Our estimates of heritability are not as high as those reported by Leaver and Taggart [28], probably because they used pooled family records instead of individual records, and because of differences in the age and diet of the fish studied.

### Effect of body weight

Our statistical model included body weight as a fixed effect, which decreased the heritability estimates, because of the high heritability of body weight. We corrected for the effect of body weight because the fish studied displayed a large variation in body weight, and body weight had a significant effect on most traits. When correcting for this trait, we removed the effect seen in Fig. 1, i.e. that the proportional contents of some FA increased or decreased with increasing body weight, which renders the relationships between these FA predictable.

### Metabolism

The additive genetic variation observed in muscle FA composition could be due to differences in the transport and deposition of these FA from the feed, or due to differences in endogenous production of these FA. When Atlantic salmon are fed diets with high levels of DHA, their endogenous capacity for bioconversion from ALA to DHA is expected to decrease because DHA is known to down-regulate the activity of the Δ6-desaturase enzyme [41]. It has also been reported that FA desaturation activity decreases with the age of the fish [18]. However, although the bioconversion capacity is relatively low, our data indicates that active bioconversion of 18:3n-3 (ALA) to 22:6n-3 (DHA) in muscle takes place in slaughter-sized salmon that are fed a high FO diet. One indicator for this active bioconversion is the negative phenotypic correlation (− 0.56) between proportional contents of ALA and DHA, the start and end-points of the pathway (Table 4). Another indicator is the positive correlations between adjacent FA in the pathway. This assumption is supported by recent studies that indicated that, although the Δ6-desaturase conversion of 18:3n-3 to 18:4n-3 is greatly inhibited in fish fed a high FO diet, the activity of the last Δ6-desaturase step of the bioconversion pathway (converting 24:5n-3 to 24:6n-3) is not lower than in fish fed a low FO diet [42, 43]. Therefore, DHA production from ALA is, to some extent, maintained [42, 43], which has been suggested to be due to an increase in the direct elongation of 18:3n-3 to 20:3n-3, bypassing the first Δ6 step (18:3n-3 to 18:4n-3). The FA 20:3n-3 is then converted to 20:4n-3, which is shuttled into the main pathway and then converted to EPA and DHA. Our detection of 20:3n-3 in the muscle further supports this assumption because 20:3n-3 is a FA that is not commonly present in significant amounts in Atlantic salmon feed. The genetic correlation of 20:3n-3 was negative with ALA and positive with DHA (Table 4), which also agrees with the above results. Although our study used large fish on a FO-rich feed, there were still indications of bioconversion influencing muscle FA composition.

Because levels of EPA and DHA in the feed are proven to affect bioconversion activity, it would be interesting to perform the same analysis on fish fed a low FO diet. Unpublished results from our group have shown that the fish with the highest conversion capacity on a high FO diet also retained this capacity when the amount of FO in the feed was reduced (personal communication Bente Ruyter). In addition, n-3 bioconversion is not expected to be the only factor that determines the FA composition of the muscle. Thus, the results of our study are relevant also for salmon in commercial production in which diets with low contents of marine ingredients are used.

### Correlations of fatty acid contents with fat deposition traits

Although our dataset of 668 fish is larger than data used in previous studies on the genetic variation of FA composition in fish (416 fish in [28] and 514 fish in [44]), only some of the genetic correlations were significant and all had large standard errors. Thus, the interpretation of the results is based on the patterns of correlations estimates across traits, rather than on individual estimates.

Individual FA appear to play different roles in the lipid metabolism, since their proportional contents differed in heritability and in correlations with fat deposition traits. In general, we found that proportional contents of all 18-carbon FA had similar correlations with the fat deposition traits, and that they followed a pattern; correlations of these FA with visceral fat and liver fat reflected those with muscle fat, and the genetic and phenotypic correlations were similar. The correlations of the saturated FA PA (16:0) with the fat deposition traits displayed opposite signs compared to the 18-carbon FA, but followed a similar pattern. However, correlations of proportional contents of EPA, DPA and DHA did not follow this pattern; their correlations with visceral fat and liver fat did not reflect their correlations with muscle fat, and the genetic correlations often differed from the phenotypic correlations.

The proportional content of EPA had favorable genetic correlations with different body fat deposits; a higher proportion of EPA in the muscle was associated with a higher amount of fat in the muscle but with less fat in liver and viscera (Table 5). The proportional content of DHA had a positive genetic correlation with visceral fat (0.61), indicating a possible genetic link between high deposition of DHA in the muscle and a high level of visceral fat (Table 5). The DHA:ALA ratio had an even higher genetic correlation with visceral fat. The basis for these correlations is unknown. Genetic correlations between proportional contents of EPA and DHA in muscle and different fat deposits for salmon fed a traditional high FO-diet have never been reported. However, feeding trials have shown that very high levels of EPA and DHA in the diet reduce the amount of visceral adipose tissue in Atlantic salmon [45], whereas very low dietary amounts of EPA and DHA are linked to fatty liver [9, 46]. Since there were no health-risk levels of liver fat in the fish of this study, the correlations of liver fat with other traits found here are not comparable to those found in feeding trials or other studies that analyzed fish with a high occurrence of health-impairing fatty liver. It should be noted that the accuracy of the visual scoring method used for liver and visceral fat is low compared to that of chemical analyses, especially of liver fat in big fish.

### Correlations of fatty acid contents with other phenotypic traits

The metabolic role of EPA may explain the correlation found between its proportional contents and sea lice density (-0.47 (0.28), Table 6), which indicated that a high proportion of EPA in muscle is associated with increased resistance to sea lice. Since the EPA content in muscle is reflected in the skin [15], this correlation may be due to the increased levels of EPA in the skin having anti-inflammatory and immunological effects [47]. The effect of EPA may be similar to that of other anti-inflammatory factors that protect Atlantic salmon against sea lice infection [48]. To date, there is no evidence that supports a possible genetic effect of EPA on sea lice resistance, but it could be of great practical importance to the industry. However, because of the large standard error of the genetic correlation estimated here, this finding requires further investigations and should be verified in salmon fed a low FO diet.

The limitations of the method used to determine liver fat must also be taken into account when considering the observed positive correlation between liver fat and PD survival, as we do not have a biological explanation for this correlation. We found a negative genetic correlation between PD survival and muscle fat, which suggests that increased levels of fat in the muscle reduce the PD survival rate. Pancreas disease is caused by a virus that triggers inflammation in pancreas, heart and skeletal muscle tissues [49]. High levels of lipids increase the amount of inflammatory factors [50], which may explain why resistance to the PD virus infection decreases.

Muscle fat had a positive genetic correlation with pigment (Table 6). This may result from the hydrophobic nature of carotenoids, which results in their transport and absorption being closely linked to the transport of FA [51, 52]. Estimates of genetic correlations between proportional content of FA with pigment reflected those of the proportional content of FA with muscle fat, except for EPA, DPA and DHA. Since the proportional content of EPA had a positive genetic correlation with muscle fat, it was expected to have also a positive correlation with pigment, but a negative correlation of -0.09 was found. Proportional contents of DPA and DHA also had negative correlations with pigment. Previous studies reported that n-3 LC-PUFA have positive effects on the pigment content of salmon fed low levels of FO [53]. The reason why we observed the opposite may be that the fish in this study were fed high levels of these FA. Highly unsaturated FA are more susceptible to oxidation [54], which can lead to degradation of carotenoids [55].

### Possible selection strategies

From a breeder’s perspective, the goal is to increase the content of healthy omega-3 FA in salmon fillets. This can be achieved in two ways: by increasing the absolute content (grams per 100 g muscle), or by increasing the proportional content (percentage of total muscle FA). The absolute content is an important trait from the perspective of human health. The heritability for absolute content was quite high for all FA, which is linked to the high heritability of muscle fat (0.46) since estimates of genetic correlations between the absolute content of FA and muscle fat were close to 1. Thus, increased absolute contents of, e.g., EPA and DHA could be achieved by selecting for muscle fat [which had positive genetic correlations with both visceral and liver fat (see Additional file 2)]. However, the salmon breeding industry considers increased levels of muscle fat as undesirable (personal communication Håvard Bakke). Since this applies for the absolute content of all FA, the following discussion is based on proportional content only.

Based on the overall analysis of the results, among all FA, the proportional content of EPA showed the most beneficial genetic correlations with other traits, since an increased proportion of EPA in the muscle was concurrent with less liver fat and visceral fat, as well as increased resistance to sea lice. Thus, increasing the proportion of this FA could be a desirable objective, but unfortunately, its heritability is relatively low (0.09).

DHA was the most abundant n-3 LC-PUFA in the muscle and had the highest heritability (0.26). Selecting for fish with a high proportion of DHA in the muscle would lead to small and lean fish, because the proportional content of DHA decreases with increasing body weight as the TAG:PL ratio increases (Fig. 1). However, our results show that there is variation in the proportional content of DHA that is independent of body weight. The proportion of DHA corrected for body weight was not genetically correlated with muscle fat content but was positively correlated with visceral fat content. Selection for higher proportional content of DHA in the muscle may lead to improved bioconversion capacity, but it may also affect other physiological mechanisms related to FA, e.g. transport, oxidation, and deposition.

Another trait relevant for selection is the DHA:ALA ratio, since it can be an indirect measure of the bioconversion of ALA to DHA. We found that it had moderate heritability (0.20) and its correlations with other traits were similar to those of proportional content of DHA, but its correlation with visceral fat was even higher (although with a high standard error). The viscera represent a natural and healthy place for the salmon to store excess energy as fat, and thus, biologically it is meaningful. However, for Atlantic salmon breeding and production companies, visceral fat represents production loss. Visceral fat content is part of the existing breeding programs and can be dealt with as for other negatively correlated traits by using selection index theory [56]. In addition, optimizing the protein-to-lipid ratio of the feed can significantly reduce visceral lipid deposition in Atlantic salmon [57, 58]. Hence, combining selection and feeding management could prevent increased amounts of visceral fat while selecting for DHA or DHA:ALA ratio. Increasing the ratio of DHA:ALA could increase the bioconversion capacity but would not necessarily increase the absolute amount of DHA in the fillet. Furthermore, beyond bioconversion capacity, several other factors and processes probably play a role in determining the FA composition of the fillet.

From a human health perspective, selective breeding should ideally result in increases of the content of both essential FAs EPA and DHA in salmon muscle. Since EPA and DHA have different physiological functions and correlations with other traits, it may be relevant to include both FA in the selection strategy for achieving a healthy end-product. The sum of EPA and DHA (EPA + DHA) showed similar estimated genetic correlations with PD survival, lice density and pigment as the intermediate of the genetic correlations of EPA and DHA with these traits. The estimate of heritability of EPA + DHA was similar to that of EPA (0.09). In spite of this relatively low heritability, our recommendation is to genetically improve the proportion of EPA + DHA in Atlantic salmon muscle, mainly because of its aforementioned human health benefits and because it has no clear detrimental effects on other traits.

Implementing selection for changes in FA composition requires costly and time-consuming chemical analyses, which make large-scale data recording challenging. New methods for predicting FA composition may provide rapid, cost-effective measurements of muscle FA composition, and thus practical implementation of selection for this trait may become more realistic in the future.

## Conclusions

We conclude that there is additive genetic variation in the content of individual FA in salmon muscle, thus changing its FA composition by selective breeding is possible. Combined, our results indicate that individual n-3 LC-PUFA play different roles in the lipid metabolism of muscle since their heritabilities and phenotypic and genetic correlations with other traits varied. Proportional contents of EPA and DHA in the muscle were linked to body fat deposition in different ways. Several observations indicated that an active bioconversion of 18:3n-3 (ALA) to 22:6n-3 (DHA) in muscle takes place in slaughter-sized salmon fed a high FO diet. Different selection strategies could be applied to increase the content of the healthy omega-3 FA in salmon muscle. We recommend selection for proportion of EPA + DHA in the muscle since both are essential FA and because such selection has no clear detrimental effects on the other traits.

## Additional files

**Additional file 1.** Mean gross fatty acid composition of the feed provided during the final seawater stage. The values are calculated on the basis of analyses of the raw materials included in the prescription of the feed (Skretting Norway).

**Additional file 2.** Distribution of proportional content of individual fatty acids in muscle of all fish in the study. The figure shows how the muscle content of individual fatty acids (in % of total FA) is approximately normally distributed for the fish studied.

**Additional file 3.** Heritability, genetic and phenotypic correlations for fat deposition traits. Genetic parameters for muscle fat, visceral fat and liver fat. Heritability on the diagonal. Phenotypic correlations in the upper triangle and genetic correlations in the lower triangle. Standard errors in brackets.

**Additional file 4.** Phenotypic and genetic correlations between absolute content of selected muscle fatty acids and muscle fat. FA g = fatty acid content in g per 100 g of muscle. r_P_ = phenotypic correlations. r_G_ = genetic correlations. Standard errors in brackets.

## References

[1] FAO (2016). The state of world fisheries and aquaculture.

[2] Ytrestøyl T, Aas TS, Åsgård T. Resource utilisation of Norwegian salmon farming in 2012 and 2013. Nofima Report 36/2014; 2014: p. 35. https://nofima.no/wp-content/uploads/2014/11/Nofima_report_resource_utilisation_Oct_2014.pdf. Accessed 20 Mar 2018.

[3] Torstensen BE, Bell JG, Rosenlund G, Henderson RJ, Graff IE, Tocher DR (2005). Tailoring of Atlantic salmon (*Salmo salar* L.) flesh lipid composition and sensory quality by replacing fish oil with a vegetable oil blend. J Agric Food Chem.

[4] Aursand M, Bleivik B, Rainuzzo JR, Jorgensen L, Mohr V (1994). Lipid distribution and composition of commercially farmed Atlantic Salmon (*Salmo salar*). J Sci Food Agric.

[5] Zhou SY, Ackman RG, Morrison C (1996). Adipocytes and lipid distribution in the muscle tissue of Atlantic salmon (*Salmo salar*). Can J Fish Aquat Sci.

[6] Polvi SM, Ackman RG (1992). Atlantic salmon (*Salmo salar*) muscle lipids and their response to alternative dietary fatty-acid sources. J Agric Food Chem.

[7] Ruiz-Lopez N, Stubhaug I, Ipharraguerre I, Rimbach G, Menoyo D (2015). Positional distribution of fatty acids in triacylglycerols and phospholipids from fillets of Atlantic salmon (*Salmo salar*) fed vegetable and fish oil blends. Mar Drugs.

[8] Tocher DR (2003). Metabolism and functions of lipids and fatty acids in teleost fish. Rev Fish Sci.

[9] Ruyter B, Moya-Falcón C, Rosenlund G, Vegusdal A (2006). Fat content and morphology of liver and intestine of Atlantic salmon (*Salmo salar*): effects of temperature and dietary soybean oil. Aquaculture.

[10] Rye M, Gjerde B (1996). Phenotypic and genetic parameters of body composition traits and flesh colour in Atlantic salmon, *Salmo salar* L.. Aquac Res.

[11] Jobling M. Manipulating Atlantic salmon, *Salmo salar*, fillet fatty acids: can we go from fish to plant and back again? University of Tromsø; 2004. https://en.uit.no/Content/352820/Manipulating%20salmon%20fillet%20fatty%20acids.pdf. Accessed 5 Sept 2017.

[12] Torstensen BE, Froyland L, Lie O (2004). Replacing dietary fish oil with increasing levels of rapeseed oil and olive oil—effects on Atlantic salmon (*Salmo salar* L.) tissue and lipoprotein lipid composition and lipogenic enzyme activities. Aquac Nutr.

[13] Henderson RJ, Sargent JR (1985). Chain-length specificities of mitochondrial and peroxisomal beta-oxidation of fatty-acids in livers of Rainbow-trout (*Salmo gairdineri*). Comp Biochem Physiol B.

[14] Bell JG, Henderson RJ, Tocher DR, Sargent JR (2004). Replacement of dietary fish oil with increasing levels of linseed oil: modification of flesh fatty acid compositions in Atlantic salmon (*Salmo salar*) using a fish oil finishing diet. Lipids.

[15] Bou M, Berge GM, Baeverfjord G, Sigholt T, Ostbye TK, Romarheim OH (2017). Requirements of n-3 very long-chain PUFA in Atlantic salmon (*Salmo salar* L.): effects of different dietary levels of EPA and DHA on fish performance and tissue composition and integrity. Br J Nutr.

[16] Brodtkorb T, Rosenlund G, Lie Ø (1997). Effects of dietary levels of 20:5n-3 and 22:6n-3 on tissue lipid composition in juvenile Atlantic salmon, *Salmo salar*, with emphasis on brain and eye. Aquac Nutr.

[17] Vegusdal A, Ostbye TK, Tran TN, Gjoen T, Ruyter B (2004). Beta-oxidation, esterification, and secretion of radiolabeled fatty acids in cultivated Atlantic salmon skeletal muscle cells. Lipids.

[18] Tocher DR, Bell JG, McGhee F, Dick JR, Fonseca-Madrigal J (2003). Effects of dietary lipid level and vegetable oil on fatty acid metabolism in Atlantic salmon (*Salmo salar* L.) over the whole production cycle. Fish Physiol Biochem.

[19] Monroig O, Tocher DR, Navarro JC (2013). Biosynthesis of polyunsaturated fatty acids in marine invertebrates: recent advances in molecular mechanisms. Mar Drugs.

[20] Sprecher H (1981). Biochemistry of essential fatty acids. Prog Lipid Res.

[21] Park WJ, Kothapalli KSD, Lawrence P, Tyburczy C, Brenna JT (2009). An alternate pathway to long-chain polyunsaturates: the FADS2 gene product delta 8-desaturates 20:2n-6 and 20:3n-3. J Lipid Res.

[22] Monroig O, Li YY, Tocher DR (2011). Delta-8 desaturation activity varies among fatty acyl desaturases of teleost fish: high activity in delta-6 desaturases of marine species. Comp Biochem Physiol B: Biochem Mol Biol.

[23] Sprecher H (2000). Metabolism of highly unsaturated n-3 and n-6 fatty acids. Biochim Biophys Acta.

[24] Codabaccus BM, Bridle AR, Nichols PD, Carter CG (2011). An extended feeding history with a stearidonic acid enriched diet from parr to smolt increases n-3 long-chain polyunsaturated fatty acids biosynthesis in white muscle and liver of Atlantic salmon (*Salmo salar* L.). Aquaculture.

[25] Fonseca-Madrigal J, Bell JG, Tocher DR (2006). Nutritional and environmental regulation of the synthesis of highly unsaturated fatty acids and of fatty-acid oxidation in Atlantic salmon (*Salmo salar* L.) enterocytes and hepatocytes. Fish Physiol Biochem.

[26] Bell JG, Pratoomyot J, Strachan F, Henderson RJ, Fontanillas R, Hebard A (2010). Growth, flesh adiposity and fatty acid composition of Atlantic salmon (*Salmo salar*) families with contrasting flesh adiposity: effects of replacement of dietary fish oil with vegetable oils. Aquaculture.

[27] Schlechtriem C, Bron JE, Tocher DR (2007). Inter-individual variation in total fatty acid compositions of flesh of Atlantic salmon smolts-fed diets containing fish oil or vegetable oil. Aquac Res.

[28] Leaver MJ, Taggart JB, Villeneuve L, Bron JE, Guy DR, Bishop SC (2011). Heritability and mechanisms of n-3 long chain polyunsaturated fatty acid deposition in the flesh of Atlantic salmon. Comp Biochem Physiol D: Genomics Proteomics.

[29] Berge GM, Østbye TKK, Kjær MA, Sonesson AK, Mørkøre T, Ruyter B. Betydning av genetisk bakgrunn og ulike nivå av omega-3-fettsyrer i fôr i tidlig livsfaser for fiskehelse, fettsyresammensetning og muskelkvalitet ved slaktestørrelse. Nofima Report 8/2015; 2015. https://brage.bibsys.no/xmlui/handle/11250/282813. Accessed 20 Mar 2018.

[30] Wold JP, Bjerke F, Mage I (2016). Automatic control of fat content in multiple batches of meat trimmings by process analytical technology. Fleischwirtschaft Int.

[31] Folkestad A, Wold JP, Rorvik KA, Tschudi J, Haugholt KH, Kolstad K (2008). Rapid and non-invasive measurements of fat and pigment concentrations in live and slaughtered Atlantic salmon (*Salmo salar* L.). Aquaculture.

[32] Mørkøre T, Åsli M, Dessen JE, Sanden KW, Bjerke MT, Hoås KG, et al. Tekstur og fett i laksefilet. Nofima Report 38/2012; 2013. https://www.nofima.no/filearchive/Rapport%2038-2012.pdf. Accessed 20 Mar 2018.

[33] Gjerde B, Ødegård J, Thorland I (2011). Estimates of genetic variation in the susceptibility of Atlantic salmon (*Salmo salar*) to the salmon louse *Lepeophtheirus salmonis*. Aquaculture.

[34] Folch J, Lees M, Sloane Stanley GH (1957). A simple method for the isolation and purification of total lipides from animal tissues. J Biol Chem.

[35] Mason ME, Waller GR (1964). Dimethoxypropane induced transesterification of fats and oils in preparation of methyl esters for gas chromatographic analysis. Anal Chem.

[36] Gilmour AR, Cullis BR, Thompson R (2009). ASReml user guide release 3.0.

[37] Henderson CR (1984). Applications of linear models in animal breeding.

[38] Sanden M, Stubhaug I, Berntssen MHG, Lie O, Torstensen BE (2011). Atlantic salmon (*Salmo salar* L.) as a net producer of long-chain marine omega-3 fatty acids. J Agric Food Chem.

[39] Glencross BD, Tocher DR, Matthew C, Bell JG (2014). Interactions between dietary docosahexaenoic acid and other long-chain polyunsaturated fatty acids on performance and fatty acid retention in post-smolt Atlantic salmon (*Salmo salar*). Fish Physiol Biochem.

[40] Ntawubizi M, Colman E, Janssens S, Raes K, Buys N, De Smet S (2010). Genetic parameters for intramuscular fatty acid composition and metabolism in pigs. J Anim Sci.

[41] Thomassen MS, Rein D, Berge GM, Ostbye TK, Ruyter B (2012). High dietary EPA does not inhibit Delta 5 and Delta 6 desaturases in Atlantic salmon (*Salmo salar* L.) fed rapeseed oil diets. Aquaculture.

[42] Bou M, Ostbye TK, Berge GM, Ruyter B (2017). EPA, DHA, and lipoic acid differentially modulate the n-3 fatty acid biosynthetic pathway in Atlantic salmon hepatocytes. Lipids.

[43] Turchini GM, Francis DS (2009). Fatty acid metabolism (desaturation, elongation and beta-oxidation) in rainbow trout fed fish oil- or linseed oil-based diets. Br J Nutr.

[44] Nguyen NH, Ponzoni RW, Yee HY, Abu-Bakar KR, Hamzah A, Khaw HL (2010). Quantitative genetic basis of fatty acid composition in the GIFT strain of Nile tilapia (*Oreochromis niloticus*) selected for high growth. Aquaculture.

[45] Todorcevic M, Kjaer MA, Djakovic N, Vegusdal A, Torstensen BE, Ruyter B (2009). N-3 HUFAs affect fat deposition, susceptibility to oxidative stress, and apoptosis in Atlantic salmon visceral adipose tissue. Comp Biochem Physiol B: Biochem Mol Biol.

[46] Liland NS, Hatlen B, Takle H, Venegas C, Espe M, Torstensen BE (2015). Including processed poultry and porcine by-products in diets high in plant ingredients reduced liver TAG in Atlantic salmon *Salmo salar* L.. Aquac Nutr.

[47] Calder PC (2006). n-3 polyunsaturated fatty acids, inflammation, and inflammatory diseases. Am J Clin Nutr.

[48] Martin SAM, Król E (2017). Nutrigenomics and immune function in fish: new insights from omics technologies. Dev Comp Immunol.

[49] McLoughlin MF, Graham DA (2007). Alphavirus infections in salmonids—a review. J Fish Dis.

[50] Berg AH, Scherer PE (2005). Adipose tissue, inflammation, and cardiovascular disease. Circ Res.

[51] Clevidence BA, Bieri JG (1993). Association of carotenoids with human plasma lipoproteins. Methods Enzymol.

[52] Bjerkeng B, Refstie S, Fjalestad KT, Storebakken T, Rødbotten M, Roem AJ (1997). Quality parameters of the flesh of Atlantic salmon (*Salmo salar*) as affected by dietary fat content and full-fat soybean meal as a partial substitute for fish meal in the diet. Aquaculture.

[53] Ytrestøyl T, Krasnov A. Lite omega-3 gir blek laks. Næringsnytte. 2016;6:24. https://nofima.no/forskning/naringsnytte/lite-omega-3-gir-blek-laks/. Accessed 10 Mar 2018.

[54] Arab-Tehrany E, Jacquot M, Gaiani C, Imran M, Desobry S, Linder M (2012). Beneficial effects and oxidative stability of omega-3 long-chain polyunsaturated fatty acids. Trends Food Sci Technol.

[55] Boon CS, McClements DJ, Weiss J, Decker EA (2010). Factors influencing the chemical stability of carotenoids in foods. Crit Rev Food Sci Nutr.

[56] Hazel LN (1943). The genetic basis for constructing selection indexes. Genetics.

[57] Dessen JE, Weihe RN, Hatlen B, Thomassen MS, Rorvik KA (2017). Different growth performance, lipid deposition, and nutrient utilization in in-season (S1) Atlantic salmon post-smolt fed isoenergetic diets differing in protein-to-lipid ratio. Aquaculture.

[58] Weihe R, Dessen JE, Arge R, Thomassen MS, Hatlen B, Rørvik KA (2018). Improving production efficiency of farmed Atlantic salmon (*Salmo salar* L.) by isoenergetic diets with increased dietary protein-to-lipid ratio. Aquac Res.

