# Supplementary material for: Genetic effects of fatty acid composition in muscle of Atlantic salmon
Source: Genet Sel Evol. 2018 May 2;50:23. doi: 10.1186/s12711-018-0394-x (PMC5932797; doi:10.1186/s12711-018-0394-x)
Supplement: Supplementary file 1 — Additional file 1. Mean gross fatty acid composition of the feed provided during the final seawater stage. The values are calculated on the basis of analyses of the raw materials included in the prescription of the feed (Skretting Norway). [file 12711_2018_394_MOESM1_ESM.docx]

|  | **% of feed** | **% of total fat** | **Ratio** |
| --- | --- | --- | --- |
| Crude fat | 36.0 |  |  |
| EPA+DHA | 6.2 | 17 |  |
| Saturated FAs | 8.5 | 23.8 |  |
| Monounsaturated FAs | 13.1 |  |  |
| Polyunsaturated FAs | 11.0 |  |  |
| Total omega-6 FAs | 2.4 |  |  |
| Total omega-3 FAs | 8.7 |  |  |
| EPA | 3.1 |  |  |
| DHA | 3.1 |  |  |
| n-6/n-3 |  |  | 0.3 |
| EPA/DHA |  |  | 1.1 |
