# Supplementary figures and images for: Genetic effects of fatty acid composition in muscle of Atlantic salmon
Source: Genet Sel Evol. 2018 May 2;50:23. doi: 10.1186/s12711-018-0394-x (PMC5932797; doi:10.1186/s12711-018-0394-x)

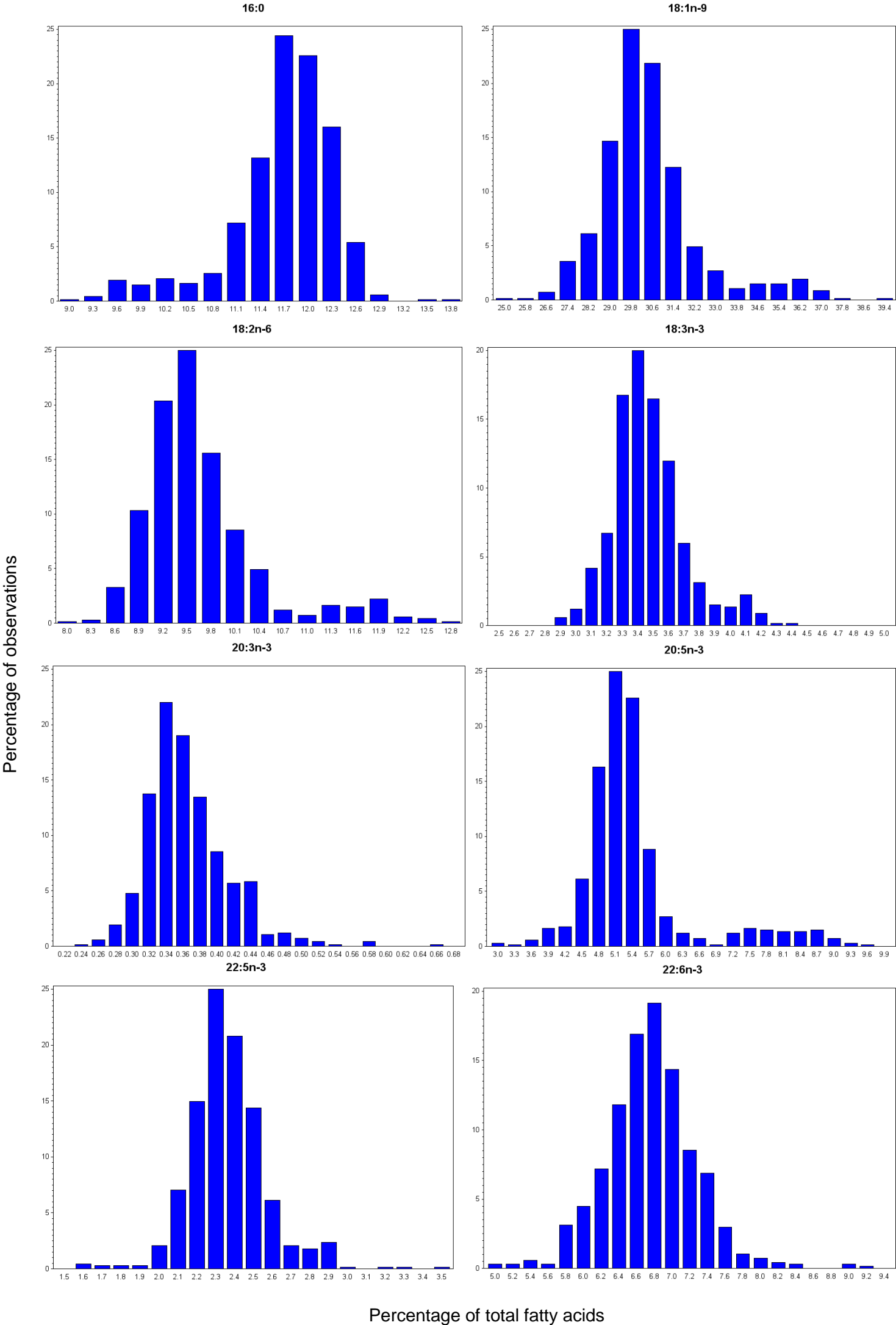

Supplement: Supplementary file 2 — Additional file 2. Distribution of proportional content of individual fatty acids in muscle of all fish in the study. The figure shows how the muscle content of individual fatty acids (in % of total FA) is approximately normally distributed for the fish studied. [file 12711_2018_394_MOESM2_ESM.pdf]
