# Supplementary material for: Genetic effects of fatty acid composition in muscle of Atlantic salmon
Source: Genet Sel Evol. 2018 May 2;50:23. doi: 10.1186/s12711-018-0394-x (PMC5932797; doi:10.1186/s12711-018-0394-x)
Supplement: Supplementary file 3 — Additional file 3. Heritability, genetic and phenotypic correlations for fat deposition traits. Genetic parameters for muscle fat, visceral fat and liver fat. Heritability on the diagonal. Phenotypic correlations in the upper triangle and genetic correlations in the lower triangle. Standard errors in brackets. [file 12711_2018_394_MOESM3_ESM.docx]

| **Trait** | **Muscle fat** | **Visceral fat** | **Liver fat** |
| --- | --- | --- | --- |
| **Muscle fat** | ***0.46 (0.10)*** | 0.32 (0.04) | 0.11 (0.04) |
| **Visceral fat** | 0.57 (0.23) | ***0.08 (0.07)*** | 0.15 (0.04) |
| **Liver fat** | 0.12 (0.24) | 0.69 (0.38) | ***0.16 (0.08)*** |
