# Supplementary material for: Genetic effects of fatty acid composition in muscle of Atlantic salmon
Source: Genet Sel Evol. 2018 May 2;50:23. doi: 10.1186/s12711-018-0394-x (PMC5932797; doi:10.1186/s12711-018-0394-x)
Supplement: Supplementary file 4 — Additional file 4. Phenotypic and genetic correlations between absolute content of selected muscle fatty acids and muscle fat. FA g = fatty acid content in g per 100 g of muscle. rP = phenotypic correlations. rG = genetic correlations. Standard errors in brackets. [file 12711_2018_394_MOESM4_ESM.docx]

| **FA (g)** | **r_P_** | **r_G_** |
| --- | --- | --- |
| **16:0** | 0.95 (0.00) | 0.99 (0.01) |
| **18:1n-9** | 0.91 (0.01) | 0.97 (0.02) |
| **18:2n-6** | 0.86 (0.01) | 0.95 (0.03) |
| **18:3n-3** | 0.90 (0.01) | 0.96 (0.03) |
| **20:5n-3** | 0.67 (0.02) | 0.96 (0.05) |
| **22:5n-3** | 0.84 (0.01) | 0.91 (0.04) |
| **22:6n-3** | 0.90 (0.01) | 0.94 (0.02) |
